# Secretome Analysis of Human Oligodendrocytes Derived from Neural Stem Cells

**DOI:** 10.1371/journal.pone.0084292

**Published:** 2014-01-02

**Authors:** Woo Kyung Kim, Deokhoon Kim, Jun Cui, Ho Hee Jang, Kwang Sei Kim, Hong Jun Lee, Seung U. Kim, Sung-Min Ahn

**Affiliations:** 1 Lee Gil Ya Cancer and Diabetes Institute, Gachon University, Incheon, Korea; 2 Asan Center for Cancer Genome Discovery, Asan Institute for Life Science, Ulsan University College of Medicine, Asan Medical Center, Seoul, Korea; 3 BRC Genome Research Center, Bio Research Complex, Incheon, Korea; 4 Department of Oncology, Ulsan University College of Medicine, Seoul, Korea; 5 Medical Research Institute, Chung-Ang University College of Medicine, Seoul, Korea; 6 Division of Neurology, Department of Medicine, UBC Hospital, University of British Columbia, Vancouver, British Columbia, Canada; University of South Florida, United States of America

## Abstract

In this study, we investigated the secretome of human oligodendrocytes (F3.Olig2 cells) generated from human neural stem cells by transduction with the gene encoding the Olig2 transcription factor. Using mRNA sequencing and protein cytokine arrays, we identified a number of biologically important secretory proteins whose expression has not been previously reported in oligodendrocytes. We found that F3.Olig2 cells secrete IL-6, PDGF-AA, GRO, GM-CSF, and M-CSF, and showed prominent expression of their corresponding receptors. Co-expression of ligands and receptors suggests that autocrine signaling loops may play important roles in both differentiation and maintenance of oligodendrocytes. We also found that F3.Olig2 cells secrete matrix metalloproteinases and matrix metalloproteinase-associated proteins associated with functional competence of oligodendrocytes. The results of our secretome analysis provide insights into the functional and molecular details of human oligodendrocytes. To the best of our knowledge, this is the first systematic analysis of the secretome of oligodendrocytes.

## Introduction

In the central nervous system (CNS), oligodendrocytes form the myelin sheath that electrically insulates axons [1], [2]. In multiple sclerosis, loss of oligodendrocytes results in demyelination and subsequent axonal degeneration [3]–[6], for which there are currently no effective therapies [7]. Despite their biological importance, oligodendrocytes have not been extensively characterized at the molecular level, partly because it is challenging to harvest oligodendrocytes from the brain, either directly or indirectly through primary culture.

Previous studies demonstrate that neural stem cells (NSCs) and oligodendrocytes prepared from NSCs can be used therapeutically [8]–[11]. The key challenges of this approach include determining how to efficiently induce NSCs to differentiate into functional oligodendrocytes and how to evaluate the functional competence of oligodendrocyte-lineage cells that have differentiated from NSCs [12].

In stem cell differentiation models, a number of secreted proteins play important roles in maintaining self-renewal or differentiation [13], [14]. Profiling all proteins secreted from a cell (i.e., the secretome) can provide insights into the functional and molecular details of that cell type. Thus, the secretomes of cells derived from stem cells may reflect the functional competence of those cells. The secretome of oligodendrocytes has not been studied in the past, due to the limited availability of purified populations of CNS cell types such as neurons, astrocytes, and oligodendrocytes. We have recently established a pure population of human oligodendrocytes generated by transducing human NSCs with the gene encoding the oligodendrocytes-specific transcription factor Olig2; the resultant cells have the characteristics of mature human oligodendrocytes [15], [16].

In this study, we characterized the secretome of human oligodendrocytes derived from human NSCs. To this end, we performed mRNA sequencing (mRNA-Seq) and quantitative analysis of the secretome using protein cytokine arrays.

## Materials and Methods

### Human NSC culture

Human NSCs were prepared from gestational week 14 human fetal brain, and then immortalized by infection with a retroviral vector encoding v-myc to generate a stable neural stem cell line, HB1.F3 (F3) [10], [12]. A retroviral vector carrying the gene encoding the Olig2 transcription factor was transduced into F3 cells, and a clonal cell line overexpressing Olig2 was established and named F3.Olig2. Stable expression of the transduced gene was confirmed by RT-PCR and immunocytochemistry [16]. Both F3 and F3.Olig2 cells were grown and maintained in Dulbecco's modified Eagle's medium with high glucose (HyClone, Logan, UT) containing 10% fetal bovine serum (FBS, HyClone) and 25 µg/mL gentamycin (Sigma-Aldrich, St Louis, MO).

### Immunocytochemistry

F3 and F3.Olig2 cells were grown on poly d-lysine-coated Aclar plastic coverslips for 3–5 days, fixed in 4% paraformaldehyde in 0.1 M phosphate buffer (pH 7.4) for 10 min, and incubated with primary antibodies overnight at 4°C, followed by visualization with fluorescent secondary antibodies (1∶500 dilution of Alexa Fluor 488 anti-mouse IgG or Alexa Fluor 594 anti-rabbit IgG; Molecular Probes, Eugene, OR) for 1 hr at RT. Images were collected using a Zeiss fluorescence microscope with an ApoTome imaging system. Cell type-specific markers employed for immunostaining were Nestin (1∶400, mouse monoclonal; Chemicon, Temecula, CA) for NSCs, Olig2 (1∶200, rabbit polyclonal; Chemicon), O4 (1∶400, mouse monoclonal IgM; Chemicon) and cyclic nucleotide phosphodiesterase (CNPase; 1∶300, mouse monoclonal; Chemicon) for oligodendrocytes, and Sox2 (1∶200, goat polyclonal; Santa Cruz Biotechnology, Santa Cruz, CA) for somatic stem cells.

### Reverse transcription and quantitative real-time PCR

Total RNA was isolated from cultured cells using the RNeasy kit (Qiagen, Valencia, CA) according to the manufacturer's directions. cDNA was synthesized with a reverse transcription kit (Qiagen). RNA samples were treated with DNase during total RNA isolation and at the cDNA synthesis step. Quantitative real-time PCR (RT-PCR) was performed in reaction mixtures containing cDNA from 30 ng reverse-transcribed RNA, 150 nM validated primer pairs, and 5 µL 2× SYBR Green ROX master mix (Life Technologies Corp., Carlsbad, CA). Quantitative RT-PCR was performed using a Prism 7900HT sequence detection system (Applied Biosystems, Foster City, CA). Evaluation of differential expression was based on the comparative Ct method (ABI Prism 7700 User Bulletin No. 2; PerkinElmer Life Sciences) using the gene encoding cyclophilin as a control. All primer sequences used in PCR experiments are listed in Table S1.

### Isolation of poly(A) RNA for mRNA-Seq

Total RNA was extracted using the TRIzol-based method (Life Technologies). After fractionation of total RNA, samples were precipitated with 0.5 M ammonium acetate, and residual salt was removed. The quality of total RNA samples was determined using an Agilent Bioanalyzer, and the samples with the greatest RNA integrity number score (score: 10) were selected for the next step. Enrichment of mRNA was performed using the oligo-dT cellulose method (Poly(A) Purist kit; Life Technologies). Isolated mRNA samples were assessed with the Bioanalyzer to confirm the enrichment of mRNA.

### mRNA-Seq

mRNA-Seq library samples were prepared using a SOLiD Total RNA-Seq kit (Life Technologies). The amount of starting RNA material for RNA fragmentation was 500 ng. RNA samples were fragmented with RNase III, hybridized, and ligated to SOLiD adapters and ligation enzymes. cDNA was synthesized using ArrayScript Reverse Transcriptase and size-selected by separation on a TBE-urea gel. After amplification of cDNA, the yield and size distribution of the amplified cDNA were assessed using the Bioanalyzer. cDNA libraries were sequenced on SOLiD 3 plus (Life Technologies) with 50-bp single-end reads for whole-transcriptome sequencing (SRA accession number: SRX370383). Sequenced reads were uniquely aligned to the UCSC reference human genome (build hg19) using the whole-transcriptome mapping module in Bioscope 1.3.1 from Life Technologies, with default settings. BAM files were converted into BED files using BamTools.

### Bioinformatics analysis

Sequenced reads were uniquely aligned to the UCSC reference human genome (build hg19) using the whole-transcriptome mapping module in Bioscope 1.3.1 from Life Technologies with default settings. BAM files were converted into bed files using BamTools. Relative gene expression levels were determined, and differentially expressed genes were identified, using DEGseq [17] with the refFlat gene annotation from the UCSC genome browser.

To compare functional profiles of the two sequencing samples, we performed gene expression analysis using the Metacore pathway-analysis software (GeneGo, San Diego, CA). We identified the ten most significantly enriched classes among pathway maps, networks, disease biomarkers, metabolic networks, and gene ontology processes. Process network analysis was performed independently to gain more information about the interactions involving unique genes or groups of similar genes. The enriched classes were sorted, and then selected by statistical significance (*P*<0.005).

The Secreted Protein Database (SPD), a web-based secreted protein database [18], was used to identify the genes that encode secreted proteins from among the differentially expressed genes identified by our mRNA-Seq analysis. These secreted proteins, including cytokines, chemokines, hormones, digestive enzymes, antibodies, and extracellular components, were collected through the bioinformatics pipeline and defined as the secretome.

### Protein cytokine arrays

Culture medium was screened through a Human Cytokine Array (RayBiotech, Norcross, GA). After F3 and F3.Olig2 cells were grown in serum-free medium for 48 hr, the supernatants were collected and centrifuged at 2000*g* for 15 min. The concentrations of supernatants were determined using the QubitProtein Assay Kit (Invitrogen, Eugene, OR). To normalize proteins among the various culture supernatants, the internal control prepared by the manufacturer was also added to the supernatant samples. Array membranes targeting 507 proteins were incubated with supernatants overnight at 4°C, and then incubated sequentially with biotin-conjugated antibodies and horseradish peroxidase (HRP)-conjugated streptavidin. The membrane-bound proteins were detected using an LAS4000 digital imaging system (GE Healthcare) and quantified using the ImageQuant TL software (GE Healthcare). The signal values from different arrays exposed to F3 and F3.Olig2 samples were normalized using the internal controls.

## Results

### Olig2-transduced NSCs express cell type-specific markers of oligodendrocytes

F3 human NSCs, which are derived from human fetal brain, can differentiate into neurons, astrocytes, and oligodendrocytes both *in vitro* and *in vivo* [10], [12], [19]–[23]. F3.Olig2 cells were generated by transducing the gene encoding Olig2, a basic helix-loop-helix transcription factor, into F3 cells [10], [15], [16]. Overexpression of Olig2 induces NSCs to differentiate into oligodendrocytes [15], [16].

The characteristics of F3 and F3.Olig2 cells have been described previously [10], [12], [15], [16], [23]. Briefly, F3 cells express Nestin and CD133, which are NSC markers, whereas F3.Olig2 cells express O4, GalC, CNPase, and MBP, which are oligodendrocyte-specific markers [24], [25]. In this study, we evaluated the key molecular features of F3 and F3.Olig2 cells and then performed downstream analyses, including mRNA-Seq and protein cytokine array analysis.

**Figure 1:**
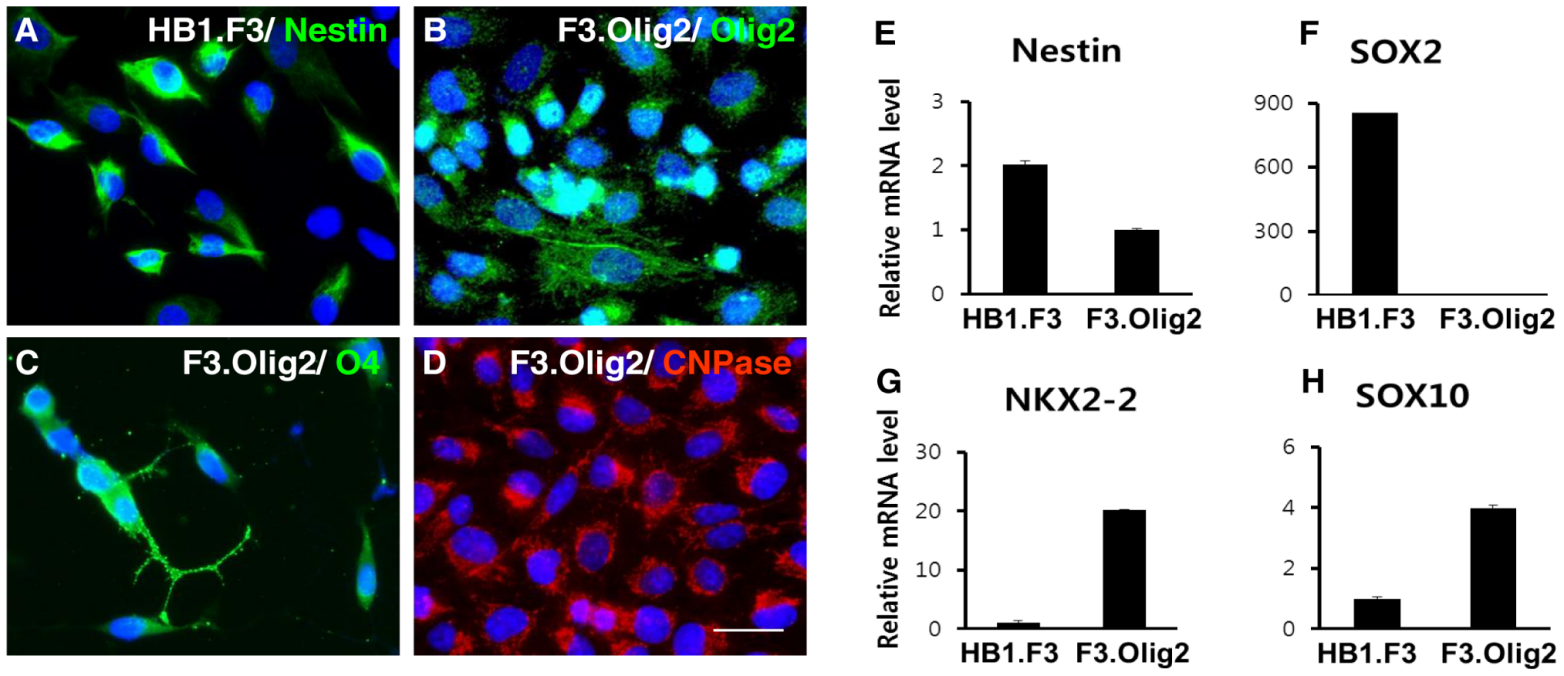
Characterization of Olig2-transduced F3.Olig2 human neural stem cells. F3.Olig2 cells and their parental cells, F3 cells, express lineage markers. (A–D) Phenotypic characterization of F3 and F3.Olig2 cells. Expression of Olig2 in nuclei of F3.Olig2 cells was confirmed through immunochemistry (Fig. 1B). Oligodendrocyte markers O4 (C) and CNPase (D) were expressed in F3.Olig2 cells, whereas F3 cells were positive for Nestin, a neural stem cell marker, and negative for Olig2, O4, and CNPase (data not shown). Nuclei were stained blue by DAPI. Scale bar, 20 µm. (E–H) Quantitative RT-PCR confirmed that the expression of the neural stem cell marker genes, Nestin and SOX2, was decreased after introduction of the OLIG2 gene. The oligodendrocyte-specific transcription factor genes NKX2-2 and SOX10 were expressed in F3.Olig2 cells. Gene expression levels were quantified relative to expression of cyclophilin.

F3 cells expressed Nestin, a NSC marker, at the mRNA and protein levels (Fig. 1A, E). F3 cells also strongly expressed of SOX2, a key transcription factor for the maintenance of NSCs in both the developing and adult brain [26], [27] (Fig. 1F). Taken together, these findings suggest that F3 cells have the general characteristics of NSCs.

In F3.Olig2 cells, transduced Olig2 was expressed and localized in nuclei (Fig. 1B). F3.Olig2 cells expressed O4 and CNPase, which are oligodendrocyte-specific markers [28], at the protein level (Fig. 1C–D). In F3.Olig2 cells, quantitative RT-PCR revealed that SOX2, a NSC maintenance factor, was downregulated, whereas NKX2.2 and SOX10, oligodendrocyte-lineage transcription factors [29], [30], were upregulated indicating that the introduction of Olig2 changed the key transcriptional regulatory circuits of HB1.F3 cells (Fig. 1F–H). However, F3.Olig2 cells did not express MBP or PLP, two myelinating oligodendrocyte markers, under common culture conditions containing 10% FBS. A previous study demonstrated that F3.Olig2 cells differentiate into mature myelinating oligodendrocytes and form myelin in demyelinated spinal cord injury models [16], suggesting that F3.Olig2 cells may require further interactions with surrounding cells and microenvironments to undergo terminal differentiation. Taken together, these findings suggest that F3.Olig2 cells have the characteristics of pre-myelinating oligodendrocytes *in vitro* [31].

### Genes that are differentially expressed between F3 and F3.Olig2 cells

**Figure 2:**
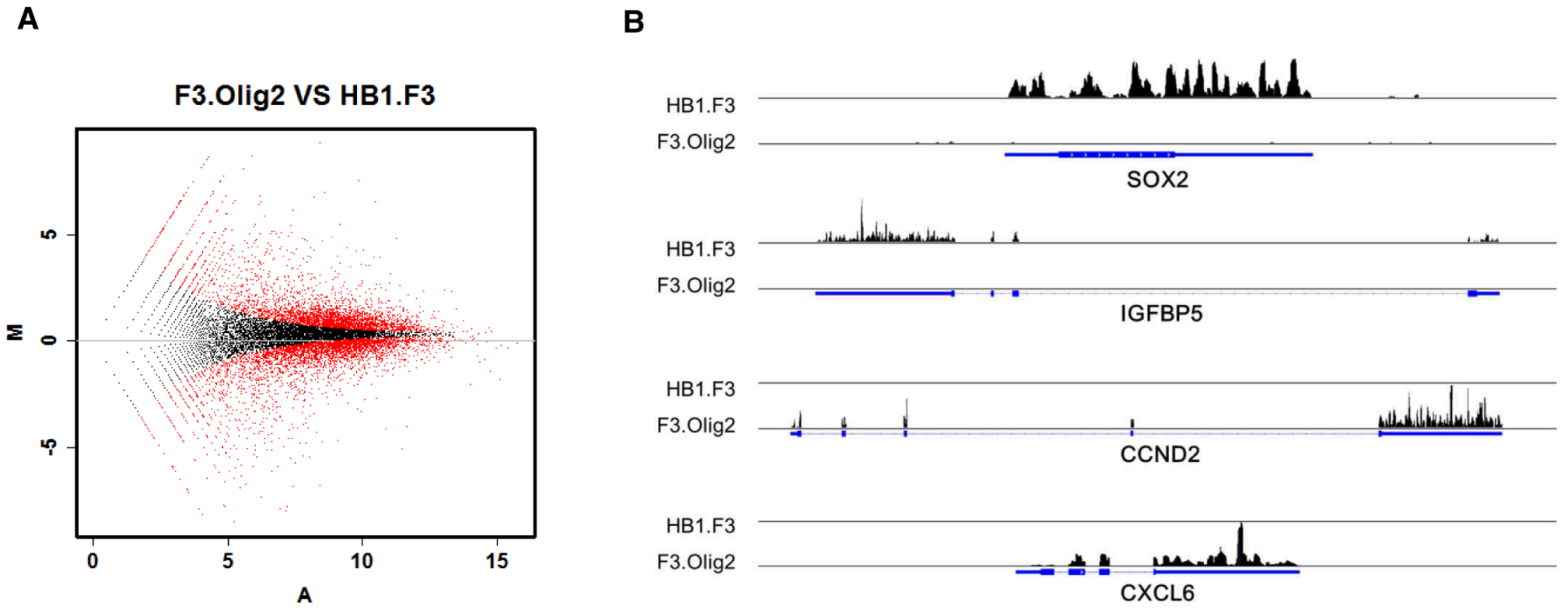
Genes expressed differentially in F3 and F3.Olig2 cells. (A) MA plot of genes (red points) shown to be differentially expressed in F3 and F3.Olig2 cells. (B) Distribution of reads mapped for differentially expressed genes in F3 (SOX2, IGFBP5) and F3.Olig2 (CCND2, CXCL6) cells, respectively.

We performed mRNA-Seq on RNAs from F3 and F3.Olig2 cells to identify genes that were differentially expressed after the introduction of Olig2 into F3 cells. In total, we obtained ∼24 and ∼25 million 50-bp sequence reads for F3 and F3.Olig2 cells, respectively. After quantitative analysis, we found that 1565 and 1286 genes were differentially upregulated in F3 cells and F3.Olig2 cells, respectively (*P*<0.001; fold-change ≥2) (Fig. 2 and Dataset S1).

**Figure 3:**
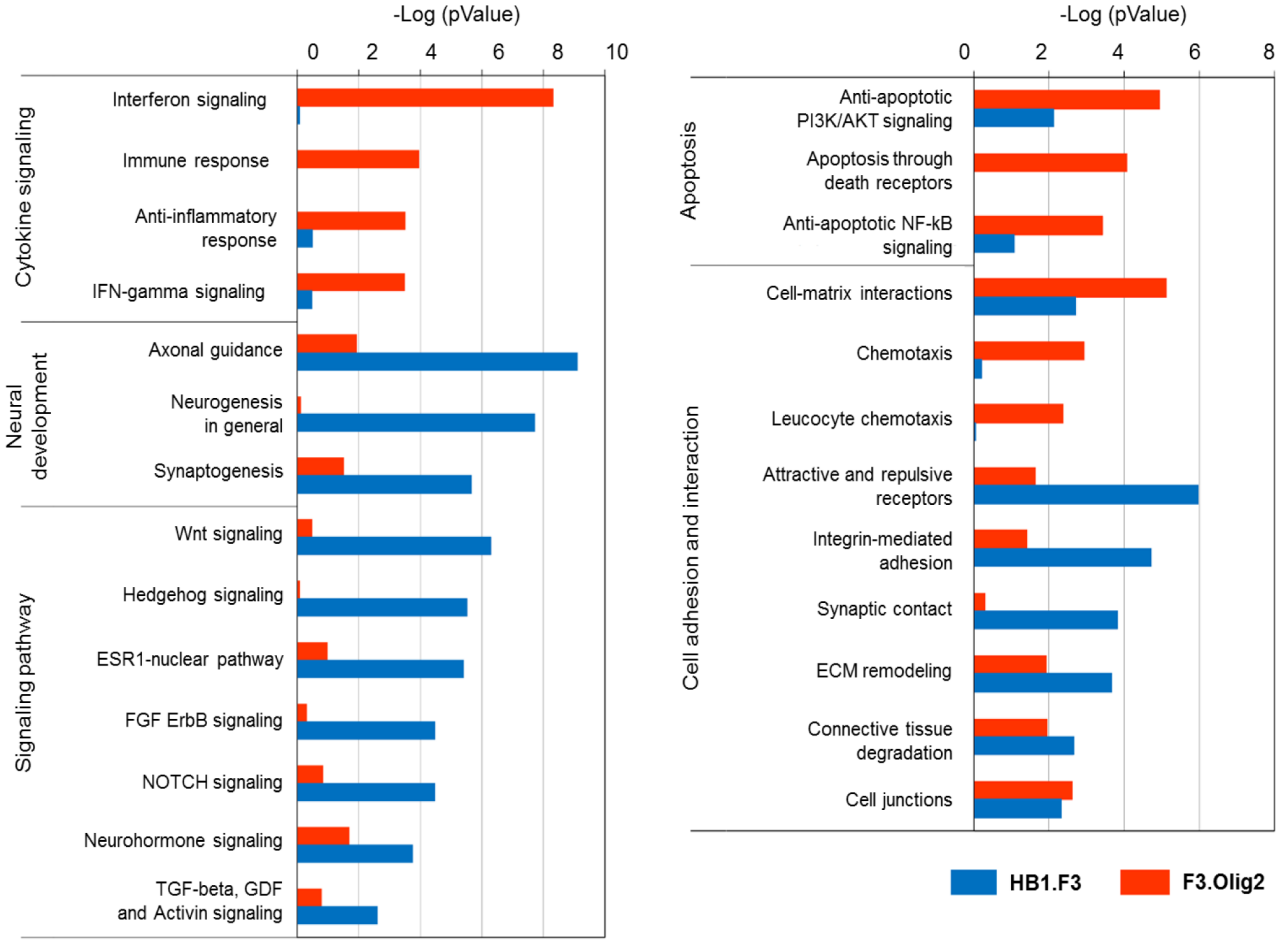
Gene-enrichment analysis of cellular processes and pathways. In F3.Olig2 cells, major cell signaling pathways, such as the Hedgehog, Wnt, and Notch signaling pathways, were downregulated, whereas cytokine signaling, chemotaxis, and cell-matrix interaction were upregulated.

Next, we performed gene enrichment analysis on differentially expressed genes to identify genetic perturbations at the pathway level. The results of gene enrichment analysis indicated that the introduction of Olig2 in F3 cells led to global gene expression changes in F3.Olig2 cells at the pathway level. Major cell signaling pathways, such as Hedgehog, Wnt, and Notch signaling, were all downregulated in F3.Olig2 cells (Fig. 3). The Hedgehog signaling pathway plays an important role in vertebrate embryonic development, such as neural tube patterning, as well as in cell fate specification, cell proliferation, and cell survival in different cell types [32]–[34]. In addition, Wnt signaling regulates the proliferation of neural progenitor cells and their differentiation into neurons in both the developing and adult brain [35], [36]. Notch signaling regulates cell fate decision and differentiation [37], [38], and exerts negative effects on oligodendrocyte differentiation that result in inhibition of myelination [39], [40].

By contrast, pathways related to cytokine signaling, chemotaxis, and cell-matrix interaction were upregulated in F3.Olig2 cells (Fig. 3, Table S2). The majority of genes involved in these pathways encode proteins that are secreted. Differentially expressed secreted signaling molecules and receptors will be further described below.

### Secreted proteins and receptors that are differentially expressed in F3.Olig2 cells

**Figure 4:**
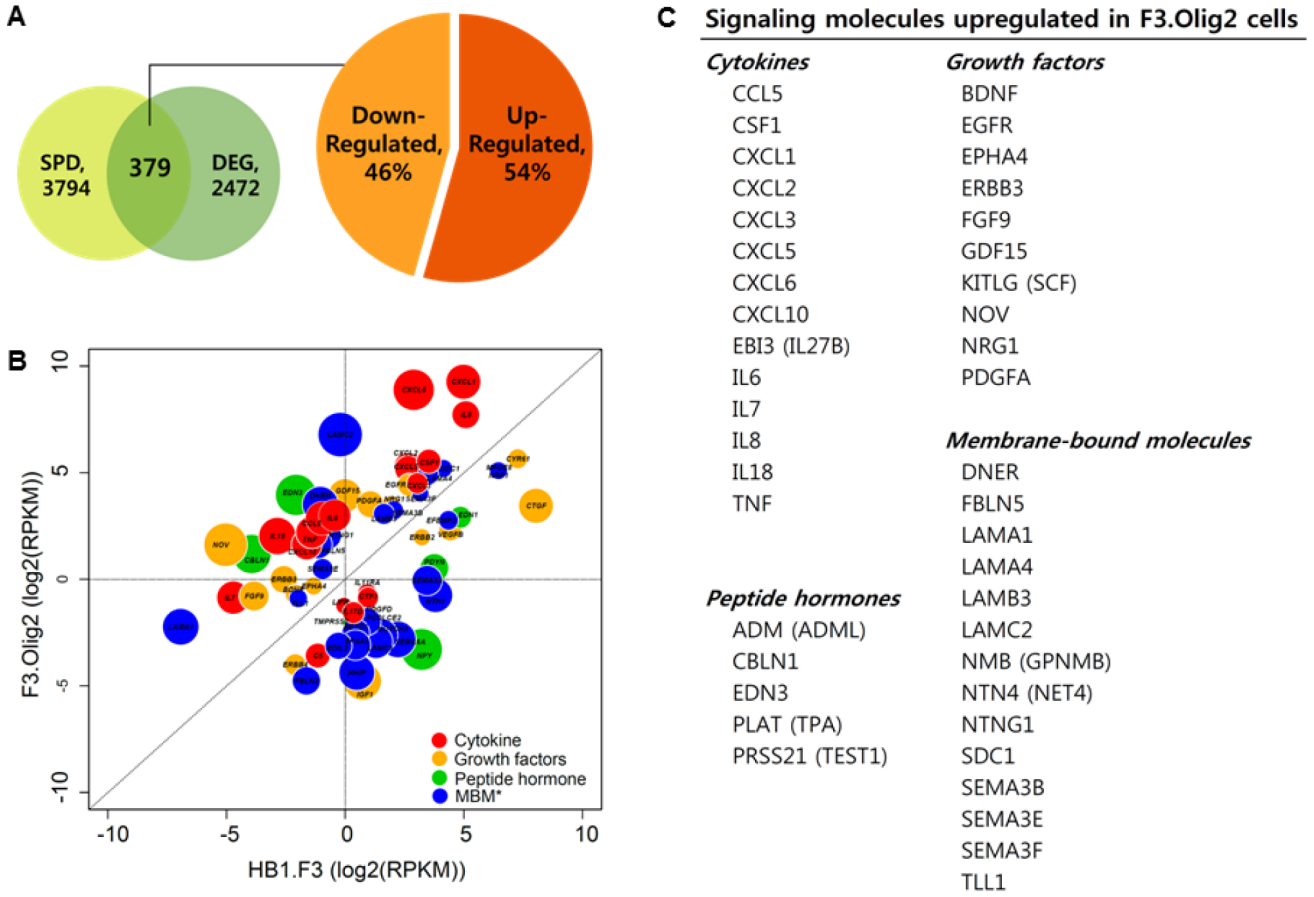
Classification of the genes that encode secreted proteins in F3.Olig2 cells. (A) A total of 379 genes that encode secreted proteins were differentially expressed in F3.Olig2 cells; 206 genes (54%) were upregulated and 173 genes (46%) were downregulated. SPD, Secreted Protein Database; DEG, Differentially Expressed Genes. (B) 206 genes, upregulated in F3.Olig2 cells, were functionally classified using the Protein ANalysis THrough Evolutionary Relationships (PANTHER) system. Bubbles in the plot represent signaling molecules expressed in HB1.F3 and F3.Olig2 cells. The size of each bubble corresponds to the absolute log2 expression ratio. Cytokines (red circles), including CXCL1, CXCL2, CXCL3, CXCL5, CXCL6, CXCL10, and CCL5, were prominently upregulated in F3.Olig2 cells. RPKM, Reads Per Kilobase per Million. *Membrane-bound molecule. (C) The list represents the genes that encode the signaling molecules, which were upregulated in F3.Olig2 cells.

To identify secreted proteins that were induced in F3.Olig2 cells, we screened the SPD for the differentially expressed genes [18] (Fig. 4A). A total of 379 genes encoding secreted proteins were differentially expressed in F3.Olig2 cells: 206 genes (54%) were upregulated and 173 genes (46%) were downregulated (Fig. 4A). Next, we investigated the functional distribution of the 206 genes that were upregulated in F3.Olig2 cells using Protein Analysis Through Evolutionary Relationships (PANTHER) [41], [42]. The ‘signaling molecule’ category accounted for the highest proportion of secreted proteins that were upregulated in F3.Olig2 cells (59/206, 27%) (Dataset S2). This category includes cytokines, growth factors, peptide hormones, and membrane-bound signaling molecules (Fig. 4B–C). Chemokines, including CXCL1 (Groα), CXCL2 (Groβ), CXCL3 (Groγ), CXCL5 (ENA-78), CXCL6 (GCP-2), CXCL10 (IP-10) and CCL5 (RANTES), were prominently upregulated in F3.Olig2 cells.

Several classes of cytokines involved in oligodendrocyte development were upregulated in F3.Olig2 cells, along with their corresponding receptors. First, IL6 and IL6 receptors such as IL6R and IL6ST were upregulated in F3.Olig2 cells. Oligodendrocytes express IL6 receptors, and the addition of external IL6/IL6R fusion protein promotes oligodendrocyte differentiation from oligodendrocyte progenitor cells (OPCs), as well as oligodendrocyte survival [43]–[45]. However, although IL-6 is expressed in rat oligodendrocytes treated with retinoic acid [46], there are no previous reports of IL6 secretion from human oligodendrocytes. Second, CXCR2 and its ligands, including CXCL1 (Groα), CXCL2 (Groβ), CXCL3 (Groγ), CXCL5 (ENA-78), CXCL6 (GCP-2), and IL8, were upregulated in F3.Olig2 cells (Fig. 4B–C), consistent with previous reports that CXCR2 is expressed in Olig2-expressing oligodendrocyte-lineage cells [47], [48] and that CXCL1 (Groα)–CXCR2 signaling promotes the proliferation of OPCs and stimulates production of MBP, a myelin protein, in cultured oligodendrocyte cell lines [49]–[51]. Third, PDGFA was upregulated in F3.Olig2 cells. PDGFA, a well-known mitogen for OPCs, maintains the number of PDGFRA-expressing OPCs in the CNS [52],[53]. F3.Olig2 cells also expressed high levels of PDGFRA, the receptor for PDGFA. In summary, we found that cytokines important for differentiation, proliferation, or survival of oligodendrocyte-lineage cells were upregulated in F3.Olig2 cells, along with their corresponding receptors. These observations suggest that these cytokines may contribute to oligodendrocyte development in an autocrine manner.

In addition to cytokines, laminins and metalloproteinases were expressed in F3.Olig2 cells. The expression levels of laminins, including LAMA4, LAMA5, LAMB1, LAMB2, LAMB3, LAMC1, and LAMC2, were high in F3.Olig2 cells. Laminins, which are major extracellular matrix proteins, are important in myelination in the CNS and peripheral nervous system (PNS) [54]–[58]. Notably in this context, studies in laminin-2-deficient animal models show that laminin-2 is essential for myelination and oligodendrocyte development [54], [59]. Laminin-2 is composed of laminin α2 (LAMA2), laminin β1 (LAMB1), and laminin γ1 (LAMC1), all of which were highly expressed in F3.Olig2 cells. The roles of LAMA4, LAMB3, and LAMC2 in the CNS have not yet been defined, and this is the first report that these factors are upregulated in oligodendrocytes. Further studies will be required to validate their biological functions in oligodendrocytes.

### Quantitative analysis of proteins secreted from F3 and F3.Olig2 cells, using cytokine arrays

**Figure 5:**
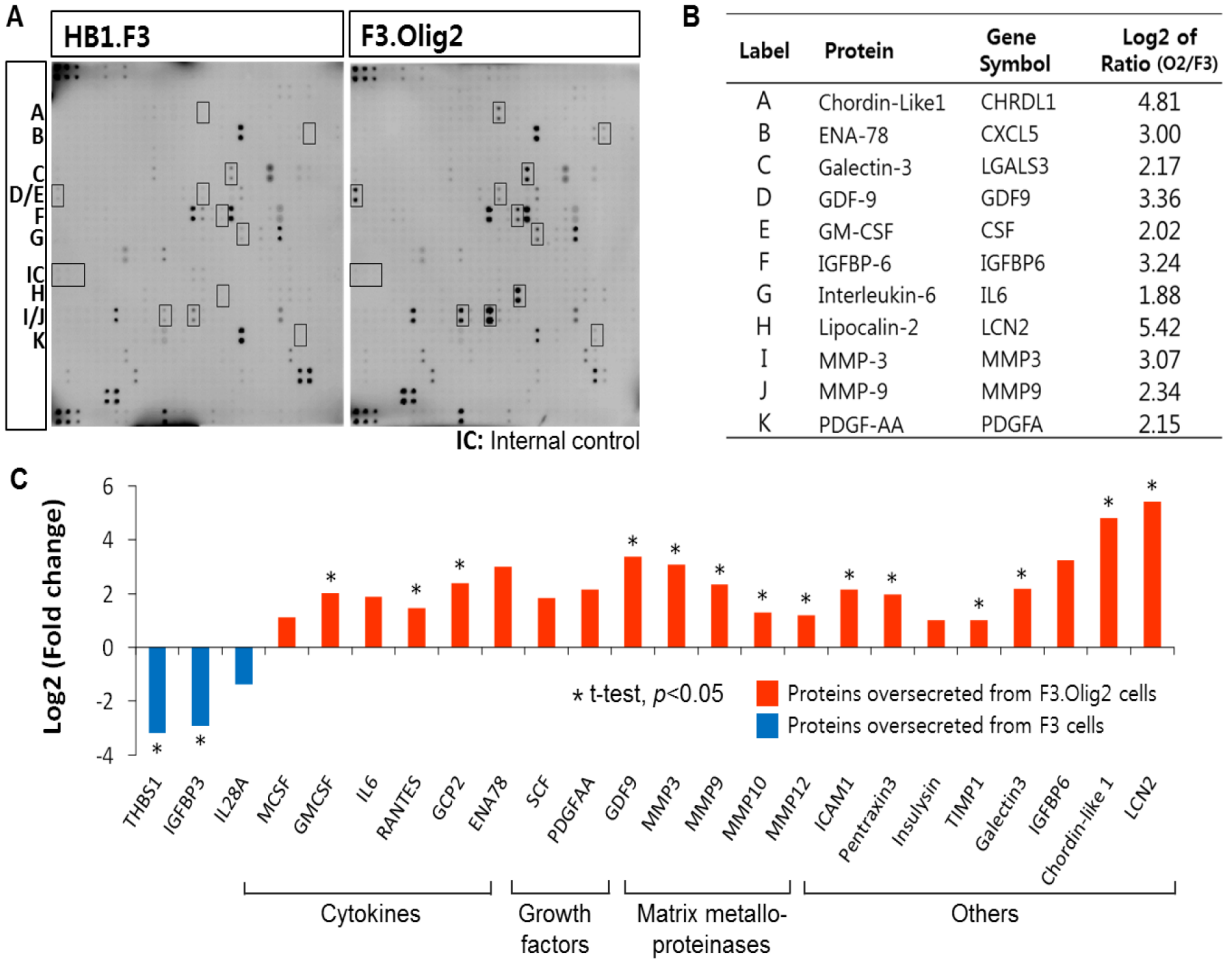
Differential secretome analysis of F3 and F3.Olig2 cells using protein cytokine arrays. (A–B) Protein cytokine arrays; top 11 proteins whose secretion was significantly increased in F3.Olig2 cells. (C) Classification of proteins which were >2-fold more abundant in supernatants from F3.Olig2 cells. Upregulation of those proteins was also confirmed at the mRNA level (data not shown).

Our analysis of secreted proteins based on mRNA-Seq data revealed that signaling molecules, including cytokines, were upregulated in F3.Olig2 cells. To validate these findings at the secreted protein level, we measured the levels of secreted proteins from F3 and F3.Olig2 cells, using a protein cytokine array targeting 507 proteins. Overall, 328 and 172 proteins were detected in the culture media of F3.Olig2 cells and F3 cells, respectively (Fig. 5), and 65 of the 328 proteins secreted from F3.Olig2 cells were >2-fold more abundant than in supernatants from F3 cells. Comparison of the mRNA-Seq data indicated that 26 of these 65 proteins were significantly upregulated in F3.Olig2 cells (Dataset S3). Proteins that were not consistent with mRNA-Seq data, but are known to be important for oligodendrocyte biology, were validated by quantitative RT-PCR.

The proteins that were upregulated at both the mRNA and secreted protein level comprised three distinct groups (Fig. 5C, Table 1). The first group includes proteins expressed in oligodendrocytes: Chordin-like 1 [60], IL-6 [46], IGFBP-6 [61], Galectin-3 [62], GRO [63], MMP-3 [64], MMP-9 [65], [66], and MMP-12 [67], [68]. Although IL-6, IGFBP-6, Galectin-3, GRO, and MMP-3 are known to be expressed at the mRNA or protein level in oligodendrocytes, this is the first report that these proteins are secreted from human oligodendrocytes. The second group includes proteins associated with, but not known to be secreted from oligodendrocytes: CXCR2 ligands (ENA-78 and GCP-2), Lipocalin-2, PDGF-AA, and TIMP-1.

**Table 1:** Classification of proteins secreted from F3.Olig2 cells.

Classification	Protein	Gene names	Additional validation	Description, limited to OL biology	Reference
A	RANTES	CCL5	R, Q	Cytokine produced from OLs by IFN γ–OL interaction	[88]
A	Chordin-like 1	CHRDL1	R, Q	BMP antagonist; OL differentiation	[60]
A	GRO	CXCL1/2/3	R, Q	CXCR2 ligand; proliferation, positioning and survival of OPCs	[49]–[51], [63], [83], [84], [89]
A	IGFBP-6	IGFBP6	R, Q	Regulation of MMP activity	[61]
A	IL-6	IL6	R, Q	OL differentiation; OL survival	[43]–[46]
A	Galectin-3	LGALS3	R, Q	Regulation of MMP activity	[62], [71]
A	MMP-3	MMP3	R, Q	NA (neuronal transmembrane protein cleavage; proinflammatory cytokine-induced expression of MMP-3/-9 in fibroblasts and epithelial cells)	[64], [90]
A	MMP-9	MMP9	R, Q	Process extension; myelin formation	[65], [66], [69], [70]
A	MMP-12	MMP12	Q	Process extension; myelin formation	[67], [68]
B	ENA-78	CXCL5	R, Q	NA (CXCR2 ligand)	[48]
B	GCP-2	CXCL6	R, Q	NA (CXCR2 ligand)	[48]
B	FGF-Basic	FGF2	R	Positive regulation of OL-lineage cell proliferation; SHH-independent OL generation; postnatal myelination	[91]–[96]
B	Lipocalin-2	LCN2	R, Q	Regulation of MMP activity	[70]
B	PDGF-AA	PDGFA	R, Q	Proliferation of OPCs and OLs	[52], [53], [83]
B	TIMP-1	TIMP1	R, Q	Regulation of MMP activity	[69], [72]
C	M-CSF	CSF1	R, Q	NA (maintenance of neural progenitor cells)	[81]
C	GM-CSF	CSF2	Q	NA (proliferation and differentiation of microglia and astrocytes; neuroprotective function in stroke models; survival of neural progenitor cells)	[77]–[79], [85], [86]
C	GDF-9	GDF9	Q	NA	
C	ICAM-1	ICAM1	R	NA	
C	Insulysin	IDE	R	NA	
C	SCF	KITLG	R	NA (survival of NSCs)	[97]
C	MMP-10	MMP10	R, Q	NA	
C	MMP-19	MMP19	R	NA	
C	Pentraxin-3	PTX3	R, Q	NA (development of DA neurons)	[82]

A: proteins expressed in oligodendrocytes; B: proteins associated with, but not known to be secreted from, oligodendrocytes; C: proteins not reported to be associated with the biology of oligodendrocytes; R: mRNA-SEQ; Q: qRT-PCR; NA: not applicable to oligodendrocyte biology; OL: oligodendrocyte; OPC: oligodendrocyte progenitor cell; ( ): reports in different cell types

Proteins that were upregulated at both the mRNA level and the secreted protein level comprised three distinct groups, indicated here as A, B, and C.

MMP-9 and MMP-12, members of the first group, are secreted from oligodendrocytes during development, and are involved in process extension of oligodendrocytes and subsequent myelin formation [65]–[68]. Galectin-3, IGFBP-6, Lipocalin-2, and TIMP-1 are associated with the activity of MMPs [62], [68]–[70]: IGFBP-6 and Galectin-3 are substrates for MMPs expressed during myelinogenesis [68], [71], whereas TIMP-1 is an inhibitor of MMPs, controlling the MMP activity [72]. Recent studies show that Schwann cells, the myelinating cells of the PNS, produce both MMP-9 and TIMP-1 in a fine balance that ultimately controls the maturation of Schwann cells and promotes myelin formation [69]. These proteins, including the MMPs, may contribute to process extension of F3.Olig2 cells.

Another member of the first group, Chordin-like 1, is a bone morphogenetic protein (BMP) antagonist. BMP signaling proteins induce astrocyte differentiation and inhibit both neurogenesis and oligodendrocyte differentiation [73]–[76]. BMP antagonists, including BAMBI-1, Chordin-like 1 and 2, and Follistatin-like 1 and 2, are required for the production of white matter-derived oligodendrocytes [60]. In addition, some members of the first two groups of proteins are biologically important in the development and maturation of oligodendrocyte-lineage cells. For example, PDGF-AA, GRO and IL-6 induce the proliferation and differentiation of oligodendrocyte-lineage cells.

The third group included proteins that are not reported to be associated with the biology of oligodendrocytes. For example, we found that GM-CSF, M-CSF, and Pentraxin-3 were secreted from F3.Olig2 cells. These three proteins are associated with differentiation or maintenance of brain-derived cells, but are not currently known to be involved in the biology of oligodendrocytes. GM-CSF is a cytokine that induces the proliferation, chemotactic migration of astrocytes and microglia [77], [78]. GM-CSF and its receptors, CSF2RA and CSF2RB, are expressed in the adult brain and found to exert a neuroprotective effect [79]. Using quantitative RT-PCR, we confirmed that F3.Olig2 cells expressed GM-CSF and its receptors, CSF2RA and CSF2RB. The observation that F3.Olig2 cells express both GM-CSF and its corresponding receptors suggests that GM-CSF may function in an autocrine manner in the maintenance and survival of oligodendrocyte-lineage cells. M-CSF is a cytokine that induces the proliferation and differentiation of macrophages [80]. M-CSF and its receptor, CSF1R, are also expressed in the developing brain, where they maintain neural progenitor cells [81]. Pentraxin-3 is expressed at high levels in mesencephalic dopaminergic neurons [82], but its functions in the neuronal differentiation process have not been clarified in detail.

## Discussion

This study was performed to characterize the secretome of F3.Olig2 cells, human oligodendrocytes derived from human NSCs, in order to gain insight into the functional and molecular details of human oligodendrocytes. Through mRNA-Seq and protein cytokine array analyses, we showed that F3.Olig2 cells secrete several classes of biologically important proteins that may play central roles in the biology of oligodendrocytes.

First, we demonstrated that IL-6, PDGF-AA, GRO, GM-CSF, and M-CSF were expressed, in F3.Olig2 cells, together with their ligands, suggesting that these cytokines may exert their effects in an autocrine manner. For example, the secretion of CXCR ligands such as GRO (CXCL1-3), ENA-78 (CXCL5), GCP-2 (CXCL6), and IL-8 (CXCL8) from F3.Olig2 cells was accompanied by the upregulation of CXCR2 in these cells. Oligodendrocyte-lineage cells express CXCR2 and respond to CXCR2 ligands [49], [50], but secretion of CXCR2 ligands by oligodendrocytes has not been previously reported. CXCR2 signaling is involved in the proliferation of oligodendrocyte-lineage cells: proliferating OPCs in the subventricular zone of the human fetal brain express both CXCL1 and CXCR2 [63], and PDGFA and CXCL1 exert synergic effects on the proliferation of rat OPCs [83]. Additionally, CXCR2 signaling is involved in the positioning of migrating OPCs [51] and inhibition of apoptosis through Bcl-2 [84].

Second, F3.Olig2 cells secrete GM-CSF and express high levels of its receptors, CSF2RA and CSF2RB. GM-CSF is not reported to be secreted from oligodendrocyte-lineage cells. It is worthy to note that GM-CSF was found to induce proliferation of fetal and adult human microglia [77] and adult simian astrocytes in culture [78]. As in astrocytes and microglia, GM-CSF might be able to induce proliferation of oligodendrocyte lineage cells. The presence of autocrine loop of GM-CSF–CSF2R signaling in oligodendrocytes has an important implication on the maintenance and survival of oligodendrocytes. CSF2R signaling exerts an anti-apoptotic effect in neural progenitor cells by upregulating Bcl-2 and Bcl-xL [85], [86].

Third, F3.Olig2 cells secrete IL-6 and express high levels of the IL6 receptors IL6R and IL6ST. IL-6 is expressed in oligodendrocytes [46], but the secretion of IL-6 from oligodendrocyte-lineage cells is not previously reported. IL-6 signaling induces the differentiation of oligodendrocyte precursor cells into oligodendrocytes and promotes their survival [43]–[45], [87]. IL-6 signaling activates the JAK-STAT signaling pathway and promotes both differentiation and survival of oligodendrocyte precursor cells [43].

In summary, the results of this study provide insights into the functional and molecular details of human oligodendrocytes. To the best of our knowledge, this is the first report of a systematic analysis of the secretome of human oligodendrocytes. Our findings suggest that autocrine signaling loops play important roles in both differentiation and maintenance of oligodendrocyte-lineage cells. In addition, we identified a number of secreted proteins that may be important for the functional competence of oligodendrocytes.

## Supporting Information

Dataset S1 **Genes that are differentially expressed from two types of cells: HB1.F3 and F3.Olig2 cells.** DEGseq was used to compare the differential expression. The data set contains the normalized expression values and P-values for all genes. Based on these two values, 2851 genes were determined as genes upregulated in HB1.F3 cells or F3.Olig2 cells.
(XLSX)

Dataset S2 **Genes encoding secreted proteins, which are identified by SPD and functionally classified using PANTHER**
(XLSX)

Dataset S3 **Secreted protein profiling using protein cytokine array and mRNA-seq data.** The dataset contains the intensity values of protein cytokine array, which were compared with differential gene expression based on DEGseq.
(ZIP)

Table S1 **List of real time PCR primers.**
(XLSX)

Table S2 **Gene enrichment analysis of differentially expressed genes.** The analysis was performed using the Metacore pathway-analysis software. Statistically significant pathways and signaling molecules were identified in HB1.F3 and F3.Olig2 cells, respectively.
(XLSX)
